# Reduced levels of circulating adhesion molecules in adolescents with early-onset psychosis

**DOI:** 10.1038/s41537-020-00112-5

**Published:** 2020-08-18

**Authors:** Kirsten Wedervang-Resell, Thor Ueland, Pål Aukrust, Svein Friis, Kirsten B. Holven, Cecilie H. Johannessen, Tove Lekva, Vera Lonning, Runar E. Smelror, Attila Szabo, Ole A. Andreassen, Anne M. Myhre, Ingrid Agartz

**Affiliations:** 1 NORMENT Center of Excellence, Institute of Clinical Medicine, University of Oslo, Oslo, Norway; 2 Division of Mental Health and Addiction, Department of Psychiatric Research and Development, Oslo University Hospital, Oslo, Norway; 3 Research Institute of Internal Medicine, Oslo University Hospital, Rikshospitalet, Oslo, Norway; 4 K.G. Jebsen Thrombosis Research and Expertise Center, University of Tromsø, Tromsø, Norway; 5 Institute of Clinical Medicine, University of Oslo, Oslo, Norway; 6 Section of Clinical Immunology and Infectious Diseases, Oslo University Hospital, Rikshospitalet, Oslo, Norway; 7 Department of Nutrition, Institute for Basic Medical Sciences, University of Oslo, PO Box 1046, Blindern, 0317 Oslo, Norway; 8 Department of Psychiatric Research, Diakonhjemmet Hospital, Oslo, Norway; 9 Child and Adolescent Psychiatry Unit, Division of Mental Health and Addiction, Institute of Clinical Medicine, University of Oslo, Oslo, Norway; 10 Centre for Psychiatric Research, Department of Clinical Neuroscience, Karolinska Institutet, Stockholm, Sweden

**Keywords:** Biomarkers, Developmental biology, Psychosis

## Abstract

It is suggested that neurodevelopmental abnormalities are involved in the disease mechanisms of psychotic disorders. Although cellular adhesion molecules (CAMs) participate in neurodevelopment, modulate blood–brain barrier permeability, and facilitate leukocyte migration, findings concerning their systemic levels in adults with psychosis are inconsistent. We examined plasma levels and mRNA expression in peripheral blood mononuclear cells (PBMCs) of selected CAMs in adolescents with early-onset psychosis (EOP) aged 12–18 years (*n* = 37) and age-matched healthy controls (HC) (*n* = 68). EOP patients exhibited significantly lower circulating levels of soluble platelet selectin (~−22%) and soluble vascular cell adhesion molecule-1 (~−14%) than HC. We found no significant associations with symptom severity. *PSEL* mRNA expression was increased in PBMCs of patients and significantly negatively correlated to duration of illness. These findings suggest a role for CAMs in the pathophysiology of psychotic disorders.

## Introduction

In the neurodevelopmental model of schizophrenia (SZ), subtle changes in brain connectivity and circuitry^1^ together with structural abnormalities^2^ are hypothesized to cause vulnerability to disease, but these issues are far from clear. Psychotic disorders with onset before 18 years, defined as early-onset psychosis disorders (EOP), provide a unique opportunity to explore disease mechanisms and the impact of disease-specific biomarkers after psychosis onset in adolescence, a sensitive neurodevelopmental phase^3–5^.

A properly regulated immune system is essential for physiological neurodevelopment and maintenance of normal brain homeostasis^6,7^. This includes balanced cell–cell adhesion, in which cell adhesion molecules (CAMs) are of major importance. CAMs contribute to early differentiation, neurite growth, synapse formation, and myelination^8^, and a subgroup of CAMs, cadherins, play a pivotal role in neuronal circuit assembly and regulation of synaptic function^9,10^. Furthermore, CAMs participate throughout life in selective recruitment of peripheral immune cells to the central nervous system (CNS). Recent findings suggest that dysregulated infiltration of the brain parenchyma by peripheral immune cells, such as T cells and antigen-presenting cells of the monocyte–macrophage lineage, may be involved in the etiology of SZ^11^. Peripheral CD4^+^ T cells are thought to be involved in CNS surveillance^12^, adult hippocampal neurogenesis, and synaptic plasticity, thereby influencing learning and social behavior, potentially reflecting that these T cells are passing the blood–brain barrier (BBB)^13–19^. Consistent with this hypothesis, several human genetic studies have identified polymorphisms in or near CAM genes that are associated with developmental neuropsychiatric disorders such as autism spectrum disorders and SZ^8,20^.

Immune-system dysregulation is suggested to contribute to the pathophysiology of psychotic disorders^11^, and the BBB, the choroid plexus, and the recently discovered specialized lymphatic system of the mammalian brain serve as important interfaces between neuronal and peripheral immune functions^21–23^. Because adhesion molecules contribute to the regulation of BBB permeability^24,25^ and facilitate leukocyte migration to meningeal spaces and hence to the cerebrospinal fluid (CSF)^21^, they could be relevant to the etiology of various psychiatric and neurological disorders. Moreover, a recent report linked SZ pathophysiology to the decreased ability of astroglia to induce regulatory T cell migration to the brain^26^. Multiple endothelial adhesion molecules provide different signals that facilitate the process of leukocyte recruitment and diapedesis. Selectins (e.g., platelet selectin [P-selectin], endothelial selectin [E-selectin], and leukocyte selectin [L-selectin]) initiate the interaction of circulating leukocytes with the endothelium, while the immunoglobulin superfamily (e.g., intercellular adhesion molecule-1 [ICAM-1], vascular cell adhesion molecule-1 [VCAM-1], and mucosal addressin cell adhesion molecule-1 [MAdCAM-1]) provide firm adhesion and other signals necessary for extravasation. In the cerebral microvasculature, tight-junction proteins (e.g. occludins, claudins, and junctional adhesion molecule-A [JAM-A]) connect adjacent endothelial cells closely at their lateral membranes to limit permeability.

Reports on circulating levels of CAMs in adults with psychosis compared with healthy controls (HC) have been inconsistent, and both higher, lower, and similar levels of ICAM-1, VCAM-1, E-, L-, and P-selectin have been reported^27–33^. Because increased levels of several of these CAMs are associated with cardiometabolic risk factors and disease in the general population^34^, differences in demographics and comorbidity combined with staging differences could explain the inconsistency in reported levels^32^. Because adolescents with EOP may have a stronger genetic component to their illness^4,35^ and lower levels of confounders because of their young age and short illness duration, analysis of this group may help to clarify whether dysregulated levels of CAMs are acquired as a result of comorbid conditions or are related to progression of psychosis. Therefore, we investigated the hypothesis that, compared with age-matched HC, adolescents with EOP exhibit alterations in circulating levels (lower or higher) of selected soluble CAMs (sCAMs) reflecting platelet, leukocyte, and endothelial cell activation (sP-selectin, sICAM-1, sVCAM-1, and sMAdCAM), soluble tight-junction proteins (sJAM-A), and soluble neuronal cadherin (sN-CAD) and that such alterations are associated with disease severity.

## Results

Table 1 shows the demographic and clinical data of the participants in this case-controlled EOP study. Patients had significantly higher body mass index (BMI) and increased levels of triglycerides (TG) and ratios of total cholesterol/high-density lipoprotein-cholesterol (TC/HDL-C) compared with HC; smoking was also more common among patients. There was no significant difference between groups in the levels of C-reactive protein (CRP), a reliable marker of systemic inflammation.

**Table 1:** Sociodemographic and clinical characteristics of EOP patients and healthy controls.

	HC (*n* = 68)	*n*	Patients (*n* = 37)	*n*	*t* test or *χ*^2^
	Mean (±SD)		Mean (±SD)		*p* value
Age (years)	16.0 (1.4)	68	16.4 (1.3)	37	0.151
Male sex, *n* (%)	33 (48)	68	12 (32)	37	0.111
BMI (kg/m^2^)	21.1 (3.1)	67	23.1 (4.9)	37	0.032
IQ	104.6 (12.5)	59	99.9 (12.5)	35	0.082
Mother’s education (years)	15.4 (2.3)	61	14.8 (2.8)	37	0.283
Smoking daily, *n* (%)	4 (6)	68	11 (30)	37	0.001
Blood measures					
TC/HDL-C ratio	2.64 (0.70)	68	3.26 (0.97)	37	0.001
TG (mmol/L)	0.62 (0.28)	68	0.96 (0.50)	37	<0.001
CRP (mg/L)	0.83 (0.77)	67	1.23 (2.12)	37	0.159
Thrombocytes (10^9^/L)	254.0 (48.6)	64	256.0 (56.6)	36	0.851
Leukocytes (10^9^/L)	5.3 (1.8)	64	5.6 (1.5)	35	0.424
Clinical measures					
CGAS			44.4 (9.3)	37	
DUP (weeks) median (Q_1_/Q_3_)			14.0 (4/52)	37	
Illness duration (years) median (Q_1_/Q_3_)			1.1 (0.7/1.9)	37	
PANSS					
Total positive score			16.2 (4.9)	37	
Total negative score			17.7 (6.8)	35	
Total general score			35.0 (8.8)	34	
CPZ			145.0 (170.8)	37	
CPZ years			8.54 (15.1)	37	
AP medicated, *n* (%)			22 (59)	37	
Currently not AP medicated, *n* (%)			15 (41)	37	
AP naive, *n* (%)			13 (35)	37	
Medication					
Aripiprazole			10		
Risperidone			3		
Quetiapine			6		
Olanzapine			2		
Clozapine			1		
Diagnosis					
Schizophrenia spectrum			19		
Affective psychosis			4		
Other psychotic disorders			14		

*HC* healthy controls, *SD* standard deviation, *BMI* body mass index, *IQ* intelligence quotient, *TC/HDL-C ratio* total cholesterol/high-density lipoprotein-cholesterol ratio, *TG* triglycerides, *CRP* C-reactive protein, *CGAS* children’s global assessment scale, *DUP* duration of untreated psychosis, *Illness duration* illness duration in years, *Q*_*1*_ first quartile (25th percentile), *Q*_*3*_ third quartile (75th percentile), *PANSS* positive and negative syndrome scale, *CPZ* current exposure to antipsychotic medication in chlorpromazine equivalents, *CPZ years* lifetime exposure to antipsychotic medication in chlorpromazine equivalents, *AP* antipsychotic medication.

### Plasma levels of soluble adhesion molecules in patients with EOP compared with HC

**Fig. 1:**
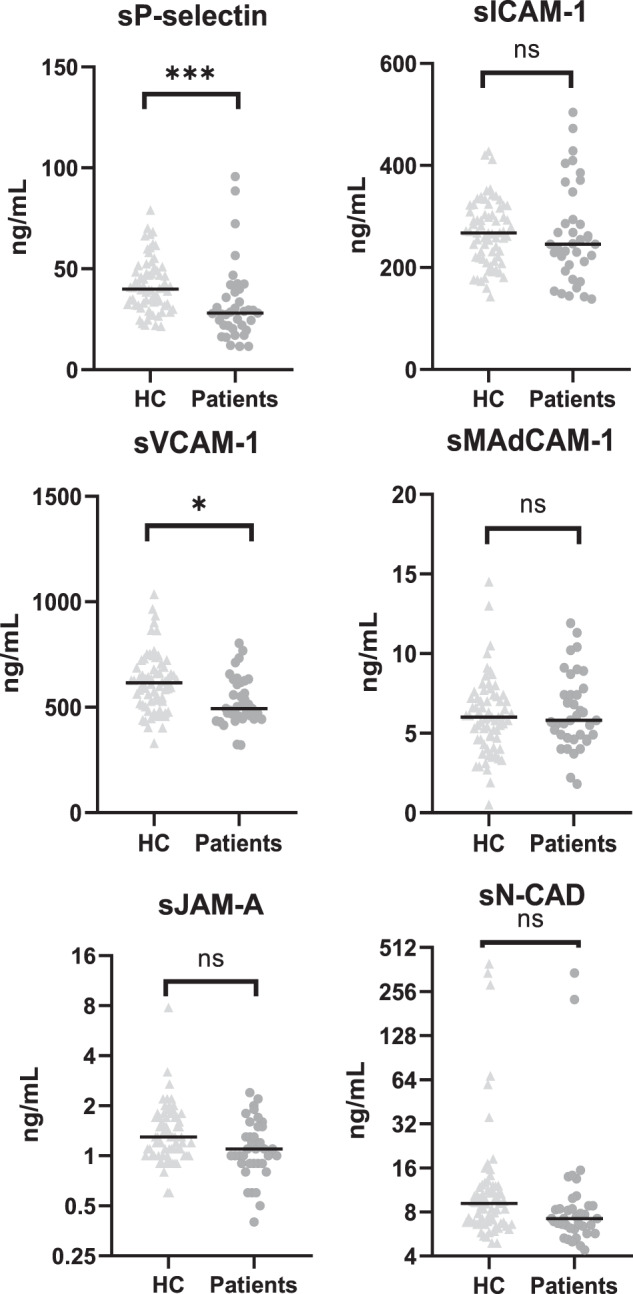
Adolescents with early-onset psychosis (*n* = 37) have reduced levels of plasma sP-selectin and sVCAM-1 compared with healthy controls (*n* = 68), whereas plasma levels of sICAM-1, sMADCAM-1, sJAM-A, and sN-CAD were not significantly different between groups, after correction for multiple testing (Bonferroni). Individual measurements are indicated as dots; the horizontal line represents the median for the whole group of patients and HC. Statistical comparison was conducted using Mann–Whitney *U* test. HC healthy controls vs. patients with early-onset psychosis, sP-selectin soluble platelet selectin, sICAM-1 soluble intercellular adhesion molecule-1, sVCAM-1 soluble vascular adhesion molecule-1, sMAdCAM-1 soluble mucosal vascular addressin cell adhesion molecule-1, sJAM-A soluble junctional adhesion molecule-A, sN-CAD soluble neuronal cadherin, ns not significant, *p < 0.05, ***p < 0.001.

To investigate any case–control differences, levels of soluble adhesion molecules were measured in fasting plasma samples from patients (*n* = 37) and HC (*n* = 68). Levels of sP-selectin (*z* = −3.96, *r* = 0.39, *p* = 0.000074) and sVCAM-1 (*z* = −3.16, *r* = 0.31, *p* = 0.002), sJAM-A (*z* = −2.33, *r* = 0.23, *p* = 0.020), and sN-CAD (*z* = −2.38, *r* = 0.23, *p* = 0.017) were significantly lower in patients than in HC. In contrast, levels of sICAM-1 (*z* = −0.95, *p* = 0.343) and sMAdCAM (*z* = 0.36, *p* = 0.717) showed no clear differences. As presented in Fig. 1, only the differences in sP-selectin and sVCAM-1 remained significant after correction for multiple testing (Bonferroni) and adjusting for age and sex. sP-selectin is released from the activated platelets, but the levels remained significantly lower in patients even after controlling for thrombocyte count.

### mRNA expression in peripheral blood mononuclear cells (PBMCs) from patients with EOP compared with HC

**Fig. 2:**
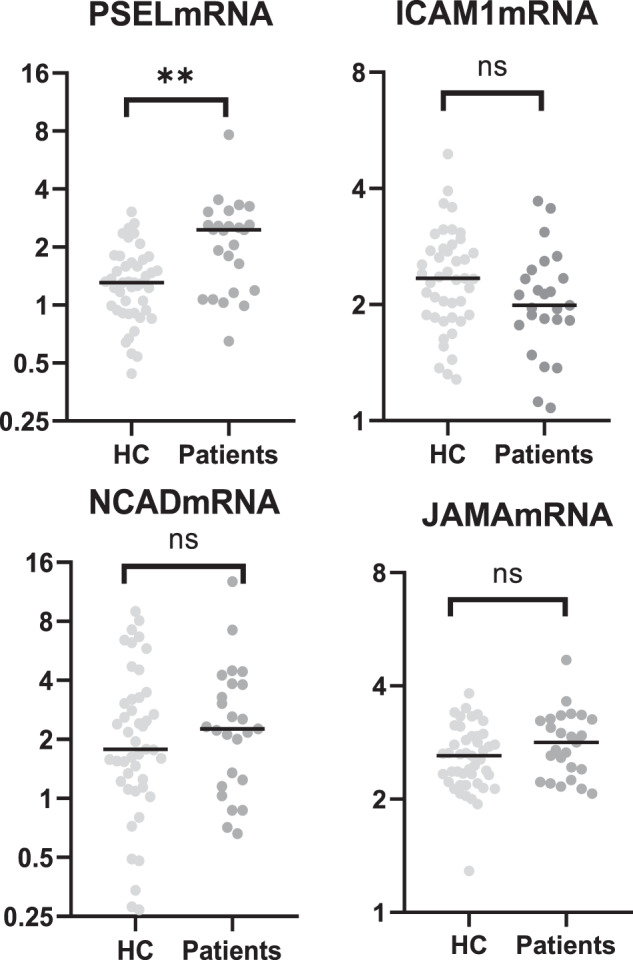
Adolescents with early-onset psychosis (*n* = 25) have increased expression levels of *PSEL* mRNA in peripheral blood mononuclear cells compared with healthy controls (*n* = 45), after correction for multiple testing (Bonferroni). The expression levels of *ICAM1* mRNA, *NCAD* mRNA, and *JAMA* mRNA showed no clear differences. Individual measurements are indicated as dots; the horizontal line represents the median for the whole group of patients and HC. mRNA levels were normalized against housekeeping genes β-actin and GAPDH and presented as relative mRNA levels on *y*-axis. Statistical comparison was conducted using Mann–Whitney *U* test. HC healthy controls vs. patients with early-onset psychosis, mRNA messenger ribonucleic acid, ICAM1 intercellular adhesion molecule-1, PSEL platelet selectin, NCAD neuronal cadherin, JAMA junctional adhesion molecule-A, ns not significant, **p < 0.01.

We investigated expression of the corresponding mRNA in PBMCs in a subsample of participants by isolating PBMCs from patients (*n* = 25) and HC (*n* = 45). Bias testing showed no significant differences in key variables between the total sample and the subsamples from whom PBMCs were collected. As presented in Fig. 2, the expression of *PSEL* mRNA in PBMCs was increased in patients compared with HC (*z* = 3.56, *r* = 0.42, *p* = 0.00038), whereas levels of *ICAM1* mRNA (*z* = −1.81, *p* = 0.071), *NCAD* mRNA (*z* = 0.576, *p* = 0.565), and *JAMA* mRNA (*z* = 1.594, *p* = 0.111) showed no significant differences. Expression of *VCAM1* mRNA and *MADCAM* mRNA was below the limit of detection. There were no significant correlations between circulating levels and the corresponding level of mRNA expression of the different adhesion molecules in PBMCs neither in the full sample nor in the subgroups (i.e., patients or HC), see Supplementary Table 1. Despite the lack of a significant correlation between the plasma and PBMC expression levels of P-selectin, both were different between the groups, suggesting existence of subgroups. Exploration of phenotypical characteristics in contrasting patient groups showed no significant differences in clinical variables, see Supplementary Table 3.

### Relationships with disease severity

We explored associations with disease severity using psychometric data (children’s global assessment score [CGAS] and positive and negative syndrome scale [PANSS] positive, negative, and general sum scores), the duration of untreated psychosis (DUP) and illness, and lifetime use of antipsychotic (AP) medication. As presented in Supplementary Table 2, there were no significant correlations between plasma levels of the adhesion molecules (sP-selectin and sVCAM-1) and markers of disease severity, whereas expression of *PSEL* mRNA in PBMCs was significantly negatively correlated with duration of illness (*r* = −0.64, *p* = 0.001).

### Role of having EOP

Post hoc regression analyses were applied to sP-selectin, sVCAM-1, and *PSEL* mRNA because these markers were significantly different in patients compared with HC after correction (Bonferroni). We evaluated whether having a psychotic disorder significantly contributed to the explained variance of sP-selectin, sVCAM-1, and PSEL mRNA after control for potential confounders (Tables 2–4). After controlling for BMI, smoking daily, TC/HDL-C ratio, and total exposure to AP medication, having EOP provided a significant additional contribution of 7.8% to the explained variance of sP-selectin, 5.0% to the explained variance of sVCAM-1, and 19.0% to the explained variance of *PSEL* mRNA expression.

**Table 2:** Total explained variance in sP-selectin.

Adjusted *R*^2^	Model 1	Model 2	Model 3	Model 4	Model 5	*p* value *R*^2^ change
BMI	–0.008					0.717
Smoking daily		0.002				0.148
TC/HDL-C ratio			–0.001			0.433
CPZ years				0.155		**<0.001**
Having EOP					0.233	**0.001**

Significant results in bold.

*sP-selectin* soluble platelet selectin, *BMI* body mass index, *TC/HDL-C ratio* total cholesterol/high-density lipoprotein-cholesterol ratio, *CPZ years* total lifetime exposure to antipsychotic medication measured in chlorpromazine equivalents, *Having EOP* having a diagnosis of early-onset psychosis.

**Table 3:** Total explained variance in sVCAM-1.

Adjusted *R*^2^	Model 1	Model 2	Model 3	Model 4	Model 5	*p* value *R*^2^ change
BMI	0.03					0.060
Smoking daily		0.03				0.205
TC/HDL-C ratio			0.02			0.915
CPZ years				0.02		0.356
Having EOP					**0.07**	**0.014**

Significant results in bold.

*sVCAM-1* soluble vascular adhesion molecule-1, *BMI* body mass index, *TC/HDL-C ratio* total cholesterol/high-density lipoprotein-cholesterol ratio, *CPZ years* total lifetime exposure to antipsychotic medication measured in chlorpromazine equivalents, *Having EOP* having a diagnosis of early-onset psychosis.

**Table 4:** Total explained variance in *PSEL* mRNA.

Adjusted *R*^2^	Model 1	Model 2	Model 3	Model 4	Model 5	*p* value *R*^2^ change
BMI	**0.05**					**0.035**
Smoking daily		0.04				0.840
TC/HDL-C ratio			0.04			0.230
CPZ years				**0.10**		**0.029**
Having EOP					**0.29**	**0.000051**

Significant results in bold.

*PSEL mRNA* platelet selectin messenger ribonucleic acid, *BMI* body mass index, *TC/HDL-C ratio* total cholesterol/high-density lipoprotein-cholesterol ratio, *CPZ years* total lifetime exposure to antipsychotic medication measured in chlorpromazine equivalents, *Having EOP* having a diagnosis of early-onset psychosis.

## Discussion

We found that adolescents with EOP exhibited reduced levels of the vascular CAMs sP-selectin and sVCAM-1, whereas *PSEL* mRNA expression was increased in PBMCs. After controlling for confounders, having EOP still explained a significant part of the difference in levels between patients and HC with 7.8% of the variance for sP-selectin, 5.0% for sVCAM-1 and 19.0% for *PSEL* mRNA expression. Levels of adhesion molecules were not correlated with markers of disease severity, including negative symptoms, but *PSEL* mRNA expression levels in PBMCs were significantly negatively correlated with duration of illness.

Adhesion molecules, BBB permeability regulation, decreased circulating levels and brain chemotaxis of regulatory T cells, and the dysfunctional control of brain immune homeostasis have been suggested as potentially important factors in the neuro-immune pathology of psychosis^26,36^. However, studies on adhesion molecules in adults with psychosis have yielded mixed results. Samples from adults with first-episode psychosis (FEP) demonstrated lower baseline levels of sVCAM-1^27,28,32^, whereas after 6 months of AP medication exposure sVCAM-1 was no longer decreased. Moreover, although Kronig et al. found decreased levels of sICAM in adults with SZ^29^, Stefanovic et al. found that early-stage SZ patients had similar levels to that of HC, and they also observed increased levels of sICAM in older patients with a longer duration of illness^32^. Increased levels of sP-selectin were shown in adult patients with acute psychosis and AP exposure^30^, whereas Mohite et al. reported no difference between HC and medicated, multi-episode patients^31^. As discussed above, these inconsistent findings could partly reflect variations in demographic characteristics between samples. This possibility is illustrated by the finding that serum levels of activated leukocyte cell adhesion molecule (sALCAM) were significantly lower in a large sample of adult patients with psychosis than in HC after control for cardiovascular disease risk factors^37^. In addition, age- and immunosenescence-related factors, differences in disease stage, disease duration, and genetic predisposition could all contribute to the different results that have been reported in relation to CAMs in psychotic disorders. In the present study, we identified lower levels of CAMs, particularly sP-selectin and sVCAM-1, in adolescents with EOP than in HC. This young population with low levels of confounders and no comorbidities such as manifest cardiovascular and metabolic disorders could represent the levels of sCAM in a “pure” psychotic disorder presenting at an early stage of disease evolution. Indeed, the observed differences appear independent of overall inflammatory activation, as reflected by low levels of CRP (a general marker of systemic inflammation) in the patients. However, as we recently demonstrated increased interleukin 18 activity in the same patients^38^, and in the current study show that the patients with shortest duration of illness have nominally higher CRP values (mean 2.6 mg/L) compared to those with longer duration, we cannot exclude involvement of immune system dysregulation in these FEP patients, which is in accordance with the overall immune hypothesis of psychosis^39^.

While soluble CAMs have multiple cellular sources, the lack of regulation of CAM mRNA levels in PBMCs and their poor correlation with plasma CAM levels suggest that the systemic downregulation of CAMs in adolescents with EOP does not merely reflect decreased expression in circulating immune cells. As such, exploration of levels of *PSEL* mRNA expression in, e.g., endothelial cells from adolescents with EOP would be of value in future studies. However, despite the lack of a significant correlation between them, both plasma and expression levels of P-selectin were different between groups. We therefore explored the possibility of subgroups by contrasting patients with combined low levels of sP-selectin and *PSEL* mRNA against those with combined high levels of sP-selectin and *PSEL* mRNA. While we did not find significant phenotypical differences between these subgroups in this small sample, the significant negative correlation between *PSEL* mRNA and duration of illness may indicate an impact of psychosis stage. Although levels of sP-selectin were reduced in the total sample of patients with EOP, sCAMs are subject to complex regulation (for instance, they increase with pro-inflammatory activation), hence, during acute psychosis expression of *PSEL* in PBMCs may be increased as part of general immune system activation^40^. This is in line with the Masopust et al. finding that markers of thrombogenesis (among others sP-selectin) were activated in an unmedicated group of adults with acute psychosis^30^. In addition, AP medications have anti-inflammatory effects, and in the present study the number of chlorpromazine (CPZ) years contributed to the total variance of sP-selectin, but not to sVCAM-1, suggesting that these effects may differ across immune markers^41,42^. However, as the current study mainly comprised FEP patients, it was impossible to investigate differences between acute and chronic stages. Nevertheless, our findings suggest that there may be subgroups with different phenotypical characteristics, related to stage (higher P-selectin and PSEL mRNA expression associated with acute psychosis) or different pathophysiological underpinnings (lower P-selectin associated with negative symptoms). This ought to be further explored in larger studies of adolescent patients at different stages of a psychotic illness, for instance, by comparing levels of sCAMs and leukocyte migration between blood and CSF.

Although the mechanisms behind these reduced levels of circulating adhesion molecules and tight-junction proteins are currently unknown, our findings may suggest that adhesion molecules play a role in the pathophysiology of psychotic disorders. This is supported by genetic evidence. The CAM and tight-junction pathways (as defined and experimentally validated using Kyoto Encyclopedia of Genes and Genomes) have been identified as showing enriched signals associated with SZ, including significant single-nucleotide polymorphisms (SNPs) in the genes for JAMA, PSEL, and ICAM^20^. With regard to the cadherin family, both disease-causing mutations and SNPs within or near cadherin genes have been associated with an increased risk of SZ, bipolar disorder, and autism spectrum disorders^10,43^. Furthermore, soluble ICAM-1 has been shown to be both secreted by^44^ and involved in the functional modulation of human astrocytes, thereby influencing the topology of the cortical microvasculature and overall neuroplasticity^45^. However, at this end the role of CAMs in the development of psychiatric disorders is still unclear, and further mechanistic studies examining among others the cellular physiology of trans-BBB communication between peripheral immune cells and glia cells in psychiatric disorders is needed^46^.

Aberrant levels of CAMs in adolescents with EOP support the neurodevelopmental hypothesis of psychotic disorders. In this regard, our findings are consistent with findings in patients with autism spectrum disorders, another type of neurodevelopmental disorder. Vascular adhesion molecules such as P-selectin, VCAM-1, and platelet endothelial cell adhesion molecule-1 were reported to be reduced in toddlers^47^, schoolchildren^48^, and adults^49,50^ with autism spectrum disorders. While the pathophysiological relevance of these findings remains unknown, the pivotal roles of CAMs in the maintenance of normal brain function suggest that dysregulated levels of CAMs may influence neurodevelopment.

Without mechanistic or longitudinal data, any interpretation of putative functional implications of the current findings is impossible. Nevertheless, theoretically CAMs are important components of the tightly regulated dynamic interface between the immune system and CNS, hence important for neurodevelopment and lifelong neuronal function. Although N-CAD has a role in restricting BBB permeability^51^, it is also required for physiological neurodevelopment and participates in synapse formation and plasticity^10,52,53^. Furthermore, while the highly regulated BBB safeguards a homeostatic environment in CNS tissue, decreased levels of tight-junction proteins may lead to increased permeability and nonselective paracellular diffusion. Interestingly, JAM-A in particular is proposed to be involved in paracellular leukocyte migration^54^. P-selectin and VCAM-1 are also proposed to be important determinants of cell trafficking between blood and meningeal spaces, providing selected leukocyte populations in CSF that differ in surface phenotype from the circulating cell population. T cells in noninflamed CSF are predominantly CD4^+^CD45RO^+^CD27^+^CD69^+^, a phenotype suggestive of a role in CNS surveillance rather than effector function^12^. It has been demonstrated that a subtle partial T cell deficit following an aberrant dynamic course with age may constitute a possible biological component in bipolar disorders, implicating an abnormal neuro-immune interaction in severe mental disorders^55^. In line, reduced levels of sP-selectin and sVCAM-1 may impact T cell trafficking with risk of imbalanced neuro-immune interaction, decreased brain immune surveillance, or, alternatively, a reduced capacity for the resolution of low-grade neuroinflammation^56^.

The present study has several strengths, including a young and clinically well-characterized patient sample, with low levels of confounders and no comorbid somatic diseases. In addition, 35% of the patients were AP naive. We also had a large representative group of well-characterized age-matched HC. Furthermore, we isolated PBMCs, making it possible to examine both peripheral circulating levels and mRNA expression of the CAMs. However, the study also had limitations. Because of its small sample size, the results must be considered preliminary, and there is a risk of type II errors, e.g., the levels of sN-CAD and sJAM-A were nominally and significantly reduced in the patients, but results did not withstand correction for multiple testing. In addition, with the possibility of subgroups related to different stages of illness, caution must be taken in any generalization of results to undifferentiated patients groups with unknown duration of illness. Furthermore, because PBMCs include several types of mononuclear cells, including lymphocytes and monocytes, differences in gene expression might also reflect differences between patients and HC in the cellular composition of the PBMC pool^57^. Although the total leukocyte count did not differ between patients and HC, we cannot exclude the influence of differences in type of cells, because differential blood counts were not available. Moreover, while alterations in the levels of CAMs may directly impact BBB permeability and recruitment of systemic immune cells through endothelial barriers, it is not known how well peripheral soluble levels correlate with levels of CAMs in the endothelial cells and in CNS tissue. This represents a major limitation, especially when considering the possible functional implications of reduced levels. Finally, we have only examined CAMs in peripheral blood and have no mechanistic data to show their role in the pathogenesis of neurodevelopment and EOP.

To conclude, we show that compared with HC, adolescents with EOP exhibit reduced circulating levels of the vascular adhesion molecules sP-selectin and sVCAM-1 after correction for multiple comparisons and confounders, whereas the neuronal adhesion molecule sN-CAD and the tight-junction protein sJAM-A were nominally reduced but did not withstand correction. The expression of *PSEL* mRNA was increased in patients and significantly negatively correlated with duration of illness, suggesting an association with acute episode of psychosis. These molecules serve important functions in neurodevelopment, BBB integrity, and homing of peripheral leukocytes to CNS. Hence, their functional relationships with psychiatric disorders, psychotic stages and the implications of altered levels should be further explored in experimental models to clarify the pathophysiological mechanisms.

## Methods

### Participants and study design

The current study is part of the ongoing longitudinal case-controlled Thematically Organized-Psychosis Study for Youth (Youth-TOP) at the University of Oslo and Oslo University Hospital, Norway. Inclusion criteria were: (1) meeting the Diagnostic and Statistical Manual of Mental Disorders diagnostic criteria for a schizophrenia spectrum disorder (SZ, schizophreniform, or schizoaffective disorder), an affective psychotic disorder (bipolar spectrum disorder, major depressive disorder with psychosis), or other psychotic disorders (psychosis not otherwise specified, delusional and brief psychotic disorders); (2) aged between 12 and 18 years; (3) able to provide written consent; (4) able to communicate in Norwegian. Exclusion criteria were: (1) an intelligence quotient (IQ) < 70, (2) previous moderate/severe head injury, (3) a diagnosis of substance-induced psychotic disorder, or (4) organic psychosis. HC aged 12–18 years from the same catchment area as the patients were randomly selected from the national population registry (www.ssb.no) and invited by letter to participate. HC were excluded if they: (1) currently met the criteria for, or had previously received treatment for, any Axis I diagnosis; (2) had an IQ < 70; (3) had a history of organic brain disease; or (4) had a previous moderate/severe head injury. The authors confirm that the study was conducted in accordance with the Declaration of Helsinki, version 2008 (sixth revision). Participation was based on informed consent, and for those aged <16 years, consent was also provided by parents or guardians. The study was approved by the Regional Ethics Committee (South-East) for Medical and Health Research Ethics (2009/691) and the Norwegian Data Protection Authority (2003/2052). For the current study, participants for whom baseline levels of adhesion molecules were available were recruited from January 2013 to October 2017, resulting in a total of 37 patients and 68 HC; PBMCs were available for 25 patients and 45 HC. Bias testing showed no significant differences in key variables between the total sample and the subsamples from whom PBMCs were collected. All were somatically healthy, without known autoimmune or endocrine diseases, none were receiving immune-modulating or immunosuppressant drugs, and there were no instances of comorbid substance abuse or dependence. At the time of blood sampling, none had symptoms of ongoing infectious disease.

### Clinical and sociodemographic assessments

The diagnostic evaluation was based on the Norwegian version of the semi-structured clinical interview of the Schedule for Affective Disorders and Schizophrenia for School-Age Children–Present and Lifetime Version^58^. Psychotic symptoms were assessed by the PANSS^59^ and global functioning was measured by the CGAS^60^. In this study, the EOP group comprise SZ spectrum, affective psychosis, and other psychotic disorders, because adolescents have greater diagnostic instability, with more frequent diagnostic change between psychotic diagnoses over time, relative to adults^61–63^. In this sample, none of the patients had previously presented with a psychosis, and all were still in their first treatment contact with the hospital, and had not experienced any remission. As such, they may be defined as having a FEP^64^. The median duration of illness was 1.1 year, but three patients had a duration >5 years, which may be considered chronic^64^. Information about the DUP, duration of illness, mother’s educational level (as a proxy measure of socioeconomic status), and smoking habits was obtained through clinical interviews. Smoking habits were dichotomized as yes/no smoking on a daily basis at the time of blood sampling. DUP was defined as the time interval in weeks with persistent symptoms qualifying for a score of ≥4 on any of the PANSS items—P1, delusions; P3, hallucinatory behavior; P5, grandiosity; P6, suspiciousness; or G9, unusual thought content—before the subject received adequate treatment for psychosis. Duration of illness was defined as time interval in years between the start of persistent symptoms qualifying for a score of ≥4 on any of the above PANSS items and time of blood draw. Participants were weighed on calibrated digital scales under standard conditions, height was measured using standard methods, and BMI (kg/m^2^) was calculated. For three patients and three HC with missing height values, we made imputations using the approximate height of an adolescent for a given age based on the 50th percentile of the Norwegian reference height/age chart^65^. One HC was neither weighed nor measured. Lifetime medical history was retrieved from medical records. Twenty-two patients were currently AP medicated, whereas 15 patients did not receive any AP medication at the time of blood sampling. Among the 15 nonmedicated patients, 13 where AP naive. Each patient’s previous and current types and doses of AP were converted to a CPZ-equivalent dose as described by Andreasen et al.^66^. Each type and dose were subsequently converted to CPZ years using the formula (CPZ in mg) × (time on dosage measured in years) and summed to provide a cumulative lifetime measure (CPZ years).

### Blood sampling, isolation of peripheral blood mononuclear cells, and biochemical measurements

Venous blood samples were drawn in the morning, after an overnight fast, separated, and the plasma fraction was stored at −80 °C until analysis for soluble adhesion molecules. Within 2 h after blood collection, PBMCs were isolated using BD Vacutainer Cell Preparation Tubes according to the manufacturer’s instructions (Becton Dickinson, San Jose, CA, USA), and stored as pellets at −80 °C until subsequent mRNA analyses. PBMC isolation required sufficient amounts of blood volume and human laboratory resources that was not always obtainable/available, resulting in a subsample (*n* = 70) with data on both plasma levels and mRNA levels. We bias tested the patient groups and HC groups by comparing key variables (age, sex, BMI, smoking daily, and the TC/HDL-C-ratio) and between the patient groups (diagnostic group, current and lifetime AP medication, DUP, total duration of illness, CGAS score, and PANSS (positive, negative, and general total sum scores). For biochemical analyses, serum was separated within 2 h. Fasting serum TC, HDL-C, and TG were analyzed at the Department of Clinical Biochemistry, Oslo University Hospital, Oslo, Norway according to standard enzymatic-colorimetric methods (Roche Diagnostics Norge AS, Oslo, Norway), and CRP was analyzed by particle-enhanced immunoturbimetry (Roche Diagnostics Norge AS, Oslo, Norway). CRP values below the quantification limit of 0.6 mg/L were treated as 0.6 mg/L in analyses. For immunoassays, blood was taken using ethylenediaminetetraacetic acid vials and plasma was isolated the next working day and stored at −80 °C, resulting in a range of 1–5 days before plasma isolation. Subsample analysis that included only blood samples in which plasma was isolated within 1–2 days yielded similar results (data not shown). Patients and HC were included consecutively with no significant difference in mean plasma storage time; furthermore, controlling for storage time did not change the results (data not shown). Plasma levels of sP-selectin, sVCAM-1, sICAM-1, sMAdCAM, sJAM-A, and sN-CAD were measured in duplicate by enzyme immunoassays (EIAs) using commercially available antibodies (R&D Systems, Minneapolis, MN, USA and SINO Biological, Eschborn, Germany) in a 384-well format using a combination of a SELMA (Jena, Germany) pipetting robot and a BioTek (Winooski, VT, USA) dispenser/washer. Absorption was read at 450 nm with wavelength correction set to 540 nm using an EIA plate reader (BioTek). Intra-assay and interassay coefficients of variation were <10% for all EIAs. Sensitivity, calculated as the readout + 3 standard deviations of the zero standard (*n* = 5), was <20 pg/mL for all CAMs except sJAM-A (42 pg/mL). Diurnal variation, evaluated in nonfasting samples taken at 12:00 noon compared with those taken at 08:00 a.m. (*n* = 6), revealed no significant effects (differences 86–109%, *p* > 0.2). Postprandial variation, evaluated by comparing nonfasting samples with fasting samples (*n* = 6), revealed no significant effects (differences 87–100%, *p* > 0.2). Stability measurements at room temperature and 4 °C, evaluated in four samples left on the bench or in the refrigerator for 2, 4, and 24 h, revealed no effects (average intra-individual coefficient of variation percentage for samples on bench or in refrigerator <10%).

### RNA isolation and real-time PCR

Extraction of total RNA was performed using TriZol (Invitrogen, Carlsbad, CA, USA) and purified using the Qiagen RNeasy Micro Kit (Qiagen, Valencia, CA, USA). Concentrations were determined by optical density readings on a Nanodrop ND-1000 spectrophotometer (Nanodrop Technologies, Wilmington, DE, USA). Reverse transcription was performed using a High-Capacity cDNA Archive Kit (Applied Biosystems, Foster City, CA, USA). mRNA quantification was performed using Perfecta SYBR Green PCR Master Mix (Quantabio, Beverly, MA, USA) and the standard curve method on an ABI Prism 7900 (Applied Biosystems). Transcript expression levels were normalized to the geometric mean of β-actin and glyceraldehyde 3-phosphate dehydrogenase (GAPDH) and presented as relative mRNA levels.

Sequence-specific intron-spanning oligonucleotide primers were designed for *ICAM1* (accession no. NM_000201.3) (forward) TGTGACCAGCCCAAGTTGTT, (reverse) AGTCCAGTACACGGTGAGGA, *VCAM1* (accession no. NM_001199834.1) (forward) GCACCACAGGCTCTTTTCCTA, (reverse) GGGACTTCCTGTCTGCATCC, *NCAD* (accession no. NM_001792.5) (forward) TCAGGCGTCTGTAGAGGCTT, (reverse) ATGCACATCCTTCGATAAGACTG, *PSEL* (accession no. NM_003005.4) (forward) TCCTCACAGCCACCTAGGAA, (reverse) TCAGGAAACAGGGTTGGTCC, *MADCAM1* (accession no. NM_130760.3) (forward) GTGCTGTTCAGGGTGACAGA, (reverse) CTGTGCAGGACGGGGATG, and *JAMA (F11R)* (accession no. NM_016946.6) (forward) GTCAAGCTCATCGTGCTTGTGC, (reverse) TGCCCGGTTCCCAATG. Transcript expression levels were normalized to the geometric mean of β-actin *(ACTB)* (accession no. NM_001101.3) (forward) AGGCACCAGGGCGTGAT, (reverse) TCGTCCCAGTTGGTGACGAT, and *GAPDH* (accession no. NM_001256799.1, NM_002046.4, NM_017008.3) (forward) CCAAGGTCATCCATGACAACTT, (reverse) AGGGGCCATCCACAGTCTT.

### Statistical analyses

Statistical analyses were conducted using IBM SPSS Statistics (version 25; IBM Corp., Armonk, NY, USA). All analyses were two tailed with the significance level set at *p* < 0.05. Data normality was assessed with Kolmogorov–Smirnov test. We used independent Student’s *t* or χ^2^ tests to investigate differences in demographic variables according to type. Because of the skewed distribution of some of the adhesion molecules (sP-selectin, sJAM-A, sN-CAD), we used nonparametric Mann–Whitney *U* test to explore differences between patients and HC. Nonparametric Spearman’s correlation analyses were used to explore the relationships of the adhesion molecules that were significantly lower in patients than in HC with markers of symptom severity. We applied hierarchical multiple linear regression analysis to assess the contribution of having EOP to the explained variance in the adhesion molecules and expression levels that were significantly different between patients and HC after correction for multiple testing (Bonferroni). Dependent variables with a skewed distribution were log-transformed in the regression analysis (sP-selectin and *PSEL* mRNA). Independent variables were identified as those with a theoretical potential for confounding and with significantly different levels between patient and HC and that correlated significantly with the dependent variable. TG and TC/HDL-C were strongly intercorrelated, therefore only TC/HDL-C was introduced into the regression model. Independent variables were entered one by one, with BMI at step 1, smoking daily at step 2, TC/HDL-C-ratio at step 3, total lifetime AP medication exposure at step 4, and having EOP at the last step, thereby controlling for the former independent variables. Preliminary analyses were conducted to ensure no violation of the assumptions of normality, linearity, multicollinearity, and homoscedasticity.

## Supplementary information

Supplementary Tables

nr-reporting-summary

## Data Availability

The data that support the findings of this study have repository at NORMENT/Oslo University Hospital. Restrictions apply to the availability of these data, which were used under license for the current study, and therefore not publicly available. Data can be made available from the authors under reasonable request and with permission of NORMENT/Oslo University Hospital, in accordance with the ethics agreements/research participants consent.
