# Supplementary material for: Reduced levels of circulating adhesion molecules in adolescents with early-onset psychosis
Source: NPJ Schizophr. 2020 Aug 18;6:20. doi: 10.1038/s41537-020-00112-5 (PMC7434772; doi:10.1038/s41537-020-00112-5)
Supplement: Supplementary file 1 — Supplementary Tables [file 41537_2020_112_MOESM1_ESM.pdf]

Supplementary Table 1. Spearman's bivariate correlation analyses between plasma levels of adhesion molecules and the corresponding mRNA expression level in PBMCs in the full sample and subsamples (patients and HC)

	PSEL mRNA with sP-selectin		*JAMA* mRNA with sJAM-A		*ICAM* mRNA with sICAM-1		*NCAD* mRNA with sN-CAD	
	r	p	r	p	r	p	r	p
All	0.12	0.326	-0.06	0.625	0.20	0.093	0.05	0.696
HC	0.20	0.184	-0.08	0.616	0.18	0.230	0.04	0.775
Patients	0.37	0.066	-0.02	0.910	0.20	0.328	0.03	0.871

Key: *PSEL* platelet selectin, sP-selectin soluble platelet selectin, *JAMA* junctional adhesion molecule-A, sJAM-A soluble junctional adhesion molecule-A, *ICAM* intercellular adhesion molecule-1, sICAM-1 soluble intercellular adhesion molecule-1 , *NCAD* neuronal cadherin , sN-CAD soluble neuronal cadherin, *mRNA* messenger ribonucleic acid, *r* Spearman's rank correlation coefficient, *p* p-value

Supplementary Table 2. Spearman's bivariate correlation analyses of sP-selectin, sVCAM-1 and *PSEL* mRNA with markers of disease severity (patients only)

	sP-selectin		sVCAM-1		*PSEL* mRNA	
	r	p	r	p	r	p
PANSS positive score	0.10	0.546	–0.03	0.860	–0.22	0.301
PANSS negative score	–0.06	0.719	–0.12	0.495	–0.35	0.100
PANSS general score	0.18	0.305	–0.21	0.221	–0.12	0.607
CGAS	–0.08	0.615	0.28	0.094	-0.07	0.735
DUP	0.10	0.545	–0.07	0.681	-0.36	0.079
Duration of illness	–0.29	0.079	0.09	0.592	**-0.64**	**0.001**
CPZ years	–0.22	0.186	–0.18	0.290	-0.33	0.107

Key: *sP-selectin* soluble platelet selectin, *sVCAM-1* soluble vascular adhesion molecule-1, *PSEL* mRNA platelet selectin messenger ribonucleic acid, *PANSS* positive and negative syndrome scale, *CGAS* children's global assessment scale, *DUP* duration of untreated psychosis, *CPZ years* lifetime exposure to antipsychotic medication in chlorpromazine equivalents, *r* Spearman's rank correlation coefficient, *p* p-value. Significant result in bold, also after correction for multippel testing (Bonferroni), (0.05/21 tests=0.002).

Supplementary Table 3. Phenotypical characteristics of contrasting patient groups identified by dichotomizing levels of sP-selectin and *PSEL* mRNA

	Low sPselectin-low mRNA n=9	High Pselectin-high mRNA n=8	p-value*
Male sex n (%)	4 (44)	1 (13)	0.149
Age	16.5 (1.6)	16.1 (1.4)	0.635
Diagnosis			0.351
SCZ spectrum	6	3	
Affective psychosis	0	1	
Other psychotic disorders	3	4	

Currently medicated n (%)	4 (44)	5 (63)	0.457
CPZ years, median (min, max)	4.3 (0.0, 59.3)	3.0 (0.0, 6.7)	0.673
DUP weeks, median (min, max)	50 (1,60)	10 (2,227)	0.277
Duration of illness years, median (min, max)	1.8 (1.1, 6.35)	0.8 (0.5, 4.5)	0.011
PANSS positive score	17.2 (4.1)	16.1 (4.8)	0.620
PANSS negative score	19.8 (6.5)	15.8 (6.0)	0.221
CGAS	42.1 (8.0)	41.4 (7.2)	0.846
BMI	21.4 (5.5)	24.9 (4.3)	0.165
Smoking daily n (%)	3 (33.3)	1 (12.5)	0.312
Trombocyte count	249.9 (50.9)	302.9 (31.5)	0.025
Leukocyte count	5.2 (1.1)	6.1 (1.4)	0.172
Ferritin	54.3 (48.6)	63.4 (68.6)	0.792
CRP	0.87 (0.47)	2.55 (4.47)	0.323

Key: SCZ schizophrenia*, CPZ years* lifetime exposure to antipsychotic medication in chlorpromazine equivalents, *DUP* duration of untreated psychosis, *PANSS* positive and negative syndrome scale*, CGAS* children's global assessment scale, *BMI* body mass index, *CRP* C-reactive protein, *p-value from t-test, Mann-U-Whitney or Chi-square, as appropriate
