# Supplementary material for: Reduced levels of circulating adhesion molecules in adolescents with early-onset psychosis
Source: NPJ Schizophr. 2020 Aug 18;6:20. doi: 10.1038/s41537-020-00112-5 (PMC7434772; doi:10.1038/s41537-020-00112-5)
Supplement: Supplementary file 2 — nr-reporting-summary [file 41537_2020_112_MOESM2_ESM.pdf]

nature research

Corresponding author(s): *Double-blind peer review submissions: write DBPR and your manuscript number here instead of author names.*

Last updated by author(s): *YYYY-MM-DD*

Reporting Summary

Nature Research wishes to improve the reproducibility of the work that we publish. This form provides structure for consistency and transparency in reporting. For further information on Nature Research policies, see Authors & Referees and the Editorial Policy Checklist.

Statistics

For all statistical analyses, confirm that the following items are present in the figure legend, table legend, main text, or Methods section.

n/a	Confirmed	
☐	☒	The exact sample size (*n*) for each experimental group/condition, given as a discrete number and unit of measurement
☐	☒	A statement on whether measurements were taken from distinct samples or whether the same sample was measured repeatedly
☐	☒	The statistical test(s) used AND whether they are one- or two-sided<br>*Only common tests should be described solely by name; describe more complex techniques in the Methods section.*
☐	☒	A description of all covariates tested
☐	☒	A description of any assumptions or corrections, such as tests of normality and adjustment for multiple comparisons
☐	☒	A full description of the statistical parameters including central tendency (e.g. means) or other basic estimates (e.g. regression coefficient) AND variation (e.g. standard deviation) or associated estimates of uncertainty (e.g. confidence intervals)
☐	☒	For null hypothesis testing, the test statistic (e.g. *F*, *t*, *r*) with confidence intervals, effect sizes, degrees of freedom and *P* value noted<br>*Give P values as exact values whenever suitable.*
☒	☐	For Bayesian analysis, information on the choice of priors and Markov chain Monte Carlo settings
☐	☒	For hierarchical and complex designs, identification of the appropriate level for tests and full reporting of outcomes
☐	☒	Estimates of effect sizes (e.g. Cohen's *d*, Pearson's *r*), indicating how they were calculated

Our web collection on statistics for biologists contains articles on many of the points above.

Software and code

Policy information about availability of computer code

Data collection	No software was used.
Data analysis	Statistical analyses were conducted using IBM SPSS statistics (version 25; IBM Corp.,Armonk,NY,USA).

For manuscripts utilizing custom algorithms or software that are central to the research but not yet described in published literature, software must be made available to editors/reviewers. We strongly encourage code deposition in a community repository (e.g. GitHub). See the Nature Research guidelines for submitting code & software for further information.

Data

Policy information about availability of data

All manuscripts must include a data availability statement. This statement should provide the following information, where applicable:

- Accession codes, unique identifiers, or web links for publicly available datasets
- A list of figures that have associated raw data
- A description of any restrictions on data availability

The data that support the findings of this study have repository at NORMENT/Oslo University Hospital. Restrictions apply to the availability of these data, which were used under license for the current study, and therefore not publicly available. Data can be made available from the authors under reasonable request and with permission of NORMENT/Oslo University Hospital, in accordance with the ethics agreements/research participants consent

Field-specific reporting

Please select the one below that is the best fit for your research. If you are not sure, read the appropriate sections before making your selection.

☒ Life sciences ☐ Behavioural & social sciences ☐ Ecological, evolutionary & environmental sciences

For a reference copy of the document with all sections, see nature.com/documents/nr-reporting-summary-flat.pdf

Life sciences study design

All studies must disclose on these points even when the disclosure is negative.

Sample size	No sample size calculation was done as this was an exploratory case-control study.
Data exclusions	No data were exluded from the analyses.
Replication	The results have not been replicated in independent samples.
Randomization	The sample was not randomized, as it was a clinical case-control study and not a clinical trial.
Blinding	Blinding was not possible as inclusion was based on a diagnostic evaluation of patients and controls prior to enrollment, however blood collection and analyses of plasma was done by lab technicians without knowledge of group affiliation.

Reporting for specific materials, systems and methods

We require information from authors about some types of materials, experimental systems and methods used in many studies. Here, indicate whether each material, system or method listed is relevant to your study. If you are not sure if a list item applies to your research, read the appropriate section before selecting a response.

Materials & experimental systems

n/a	Involved in the study	
☐	☒	Antibodies
☒	☐	Eukaryotic cell lines
☒	☐	Palaeontology
☒	☐	Animals and other organisms
☐	☒	Human research participants
☐	☒	Clinical data

Methods

n/a	Involved in the study	
☒	☐	ChIP-seq
☒	☐	Flow cytometry
☒	☐	MRI-based neuroimaging

Antibodies

Antibodies used	RnD systems: R&D Systems, Inc.<br>614 McKinley Place NE<br>Minneapolis, MN 55413<br>Toll Free USA, Canada: (800) 343-7475<br>VCAM1: DY809<br>ICAM1: DY720<br>P-selectin: DY137<br>MADCAM: DY6056-05<br>NCAD:DY138805<br><br>SINO biological, Düsseldorfer Str. 40<br>65760 Eschborn, Germany<br>JAM-A: SEKA10198
Validation	N/A

Human research participants

Policy information about studies involving human research participants

Population characteristics	Participants with early-onset psychosis (n=37) and healthy controls (n=68) were enrolled, with 32% males and a mean age of 16.4 years among patients compared to 48 % males and mean age of 16 among controls. Among patients the distribution of the

	DSM-IV verified diagnoses were; schizophrenia spectrum (n=19), affective psychosis (n=4), psychosis not otherwise specified (n=14), and a total of 35 % were anti-psychotic-naïve.
Recruitment	All participants were part of the ongoing longitudinal case-controlled Thematically-Organized-Psychosis Study for Youth (Youth-TOP), at the University of Oslo and Oslo University Hospital, part of the TOP research group, Norwegian Centre for Mental Disorder Research, Norway. Patients with an established or suspected diagnosis of psychosis were recruited from child and adolescent psychiatric in- and outpatient units at hospitals in Oslo and Akershus counties, Norway. They were initially assessed with the Positive and Negative Syndrome Scale (PANSS) (Kay et al., 1987) and from a review of their medical records. Those with a clear history of psychosis and/or reaching thresholds of current psychotic symptoms were diagnosed further. Healthy controls (HC) aged 12–18 years were recruited from the same catchment area as the patients, randomly drawn from the national population registry (www.ssb.no), and invited by letter to participate. Because of the study design, we cannot rule out the possibility that some patients with psychosis were never given the opportunity to participate in the study, possibly because they were considered too ill to participate. This may imply a disproportionate lower inclusion of more severely ill patients. However, if we had been able to include the more severely ill, we expect this would increase rather than decrease the differences between patients and HC.
Ethics oversight	The study was approved by the Regional Ethics Committee (South-East) for Medical and Health Research Ethics (2009/691) and the Norwegian Data Protection Authority (2003/2052).

Note that full information on the approval of the study protocol must also be provided in the manuscript.

Clinical data

Policy information about clinical studies

All manuscripts should comply with the ICMJE guidelines for publication of clinical research and a completed CONSORT checklist must be included with all submissions.

Clinical trial registration	This was not a clinical trial. It was a case control investigation.
Study protocol	Available from corresponding author on request.
Data collection	Participants for the current study were enrolled between january 2013 and october 2017.
Outcomes	Primary outcome measures (sICAM-1,sVCAM-1, sP-selectin, sNCAD, sJAMA, sMAdCAM) was measured in plasma by enzyme immunoassays.
